# Semi-permeable polymer vesicle-based prooxidative and lactate-depleting nanoreactors with sustained activity against pancreatic cancer

**DOI:** 10.3389/fbioe.2025.1626927

**Published:** 2025-06-26

**Authors:** Lili Sun, Qian Pan, Yunfei Li, Yanxi Mu, Yusheng Cheng, Yeqian Feng, Masaru Tanaka, Panyue Wen, Xianling Liu

**Affiliations:** ^1^ Department of Oncology, The Second Xiangya Hospital, Central South University, Changsha, Hunan, China; ^2^ Institute for Materials Chemistry and Engineering, Kyushu University, Fukuoka, Japan; ^3^ Department of General Surgery, Department of Biotherapy, Lanzhou University Second Hospital, Lanzhou, China; ^4^ Department of Oncology, Guilin Hospital of the Second Xiangya Hospital, Central South University, Guilin, China

**Keywords:** polymer vesicles, semipermeable polymersomes, nanoreactors, enzyme delivery, sustained activity, prooxidation, lactate depletion, immunogenic cell death

## Abstract

Polymer vesicles, also known as polymersomes, consist of polymer membranes enclosing an aqueous core and have attracted significant interest for biomedical applications. The aqueous core is particularly advantageous for encapsulating and stabilizing fragile cargo, such as proteins, to maintain long-term activity. Among these, enzyme-encapsulated polymersomes function as therapeutic nanoreactors and have gained increasing attention in recent years, especially for cancer treatment. A critical factor in their catalytic performance is ensuring semipermeability of the membrane, allowing selective exchange of small-molecule substrates while maintaining stable enzyme encapsulation. However, achieving a balance between prolonged structural integrity and optimal permeability to sustain catalytic activity remains a challenge. Here, we present oxidation-sensitive polyion complex vesicles (PICsomes) encapsulating lactate oxidase as prooxidative and lactate-depleting nanoreactors. The membrane’s built-in semipermeability and crosslinked network contribute to the prolonged enzymatic activity of lactate oxidase. Notably, in response to reactive oxygen species (ROS), the nanoreactors undergo swelling, further enhancing membrane permeability to amplify enzymatic catalysis—specifically, ROS production and lactate depletion. This self-amplifying function enhances cytotoxic effects against pancreatic cancer cells. Interestingly, the prooxidative activity also induces immunogenic cell death, as evidenced by elevated levels of calreticulin and HMGB1, suggesting the potential to stimulate antitumor immunity. It is important to note that lactate not only serves as a key respiratory fuel but also facilitates immune evasion. Given these findings, the reported nanoreactors offer a promising strategy for disrupting tumor energy and redox metabolism through lactate depletion and prooxidation, while also priming antitumor immunity for combination immunotherapy.

## 1 Introduction

Polymer vesicles, commonly referred to as polymersomes, are self-organized amphiphilic block copolymer nanostructures, forming a membrane surrounding an aqueous core (Vanhest et al., 1995; Zhang and Eisenberg, 1995; Discher et al., 1999; Discher and Eisenberg, 2002). These vesicles have garnered significant interest in biomedical applications, particularly in drug delivery and nanoreactor-based therapies, due to their tunable size, chemical versatility, and ability to encapsulate both hydrophilic and hydrophobic agents. A major benefit of polymersomes lies in their water-filled interior, which provides a protective microenvironment for fragile biomolecules such as proteins and enzymes, allowing for sustained bioactivity. As a result, enzyme-loaded polymersomes have been extensively explored as catalytic nanoreactors, offering potential in a wide range of applications, including biocatalysis, biosensing, and therapeutic interventions (Brinkhuis et al., 2011; Tanner et al., 2011; Balasubramanian et al., 2016; Palivan et al., 2016; Zhu et al., 2017; Pottanam Chali and Ravoo, 2020).

Among enzyme-loaded polymersomes, their application in cancer therapy has attracted growing attention (Discher et al., 2002; Napoli et al., 2004a; Napoli et al., 2004b; Ranquin et al., 2005; Koide et al., 2006; Anraku et al., 2016; Deng et al., 2016; Li et al., 2017a; Li et al., 2017b; Nishimura et al., 2017; Sueyoshi et al., 2017; Li et al., 2020; Japir et al., 2021). Enzymatic nanoreactors are designed to catalyze specific biochemical reactions either within tumors or systemically, modulating metabolic pathways to disrupt cancer cell survival. A crucial factor in the effectiveness of these nanoreactors is membrane semipermeability, which governs the controlled exchange of reactants and products while ensuring enzyme retention. However, a major challenge in the design of enzyme-loaded polymersomes is striking a balance between maintaining prolonged structural integrity and optimizing membrane permeability to sustain catalytic activity (Discher et al., 2002; Gräfe et al., 2014; Wang et al., 2022). Overcoming this challenge is essential for ensuring prolonged therapeutic effects and enhancing anticancer efficacy.

**FIGURE 1:**
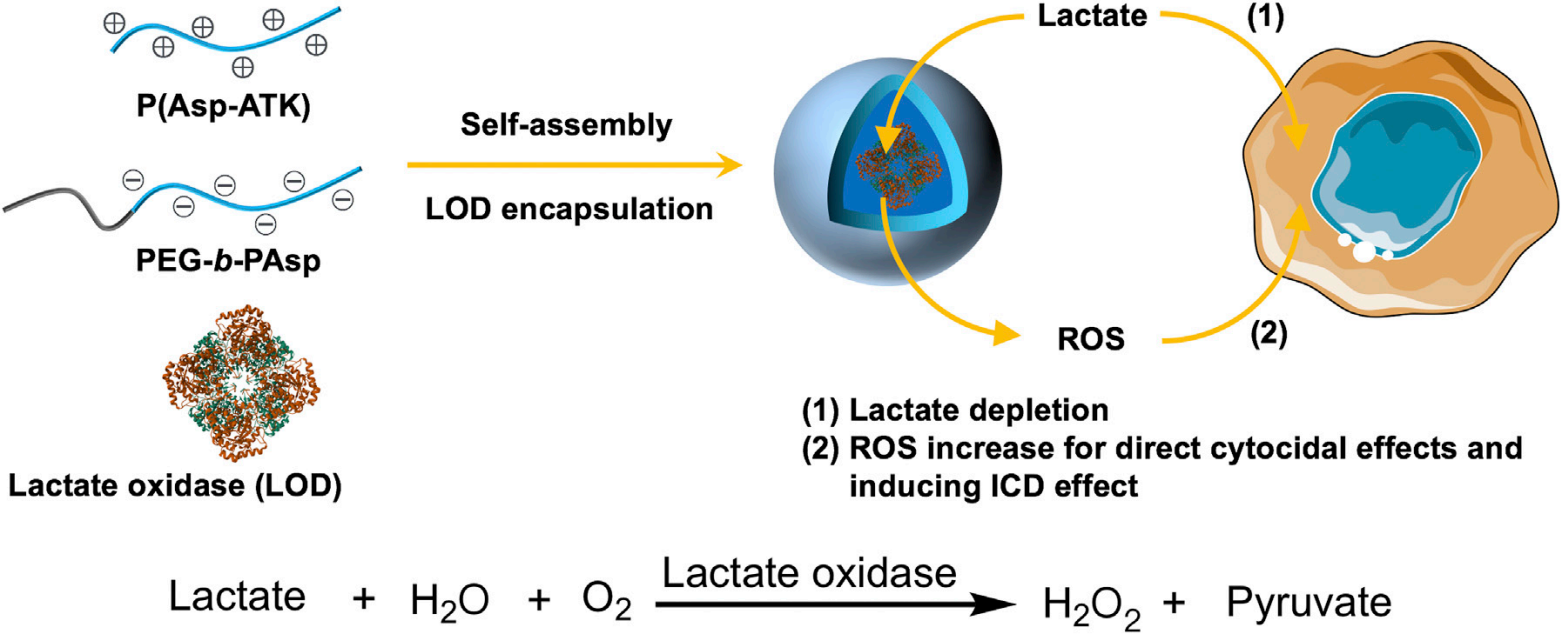
Schematic illustration of the construction of lactate oxidase (LOD)-loaded PICsomes as prooxidative and lactate-depleting nanoreactors with potent cytocidal effects. The self-assembly process occurs via electrostatic complexation between oppositely charged P(Asp-ATK) and PEG-*b*-PAsp in the presence of LOD.

In this study, we present a class of reactive oxygen species (ROS)-responsive polyion complex vesicles (PICsomes) encapsulating lactate oxidase (LOD) as prooxidative and lactate-depleting nanoreactors (Figure 1). The design of these PICsomes leverages their semipermeability and crosslinked membrane structure, which contribute to the prolonged enzymatic activity of LOD (Anraku et al., 2016; Sueyoshi et al., 2017; Li et al., 2020; Wen et al., 2025). Furthermore, in response to elevated ROS levels, the PICsomes undergo structural swelling, which enhances membrane permeability, thereby amplifying the enzymatic catalysis of lactate, producing hydrogen peroxide (H_2_O_2_). This self-amplifying mechanism results in increased oxidative stress and lactate depletion within the tumor, leading to enhanced cytotoxic effects on pancreatic cancer cells.

Pancreatic carcinoma persists as one of the most treatment-refractory and deadly cancers, characterized by a highly immunosuppressive microenvironment and metabolic adaptations that promote tumor progression (Neoptolemos et al., 2018; Bear et al., 2020; Huber et al., 2020; De Santis et al., 2024; Hu and O’Reilly, 2024). Lactate, a byproduct of glycolysis, plays a pivotal role in tumor metabolism by serving as a critical respiratory fuel and by contributing to immune evasion through the suppression of antitumor immune responses. Strategies aimed at disrupting lactate metabolism have emerged as a promising avenue for pancreatic cancer treatment (Habrook and Lyssiotis, 2017; Qin et al., 2020; Rabinowitz and Enerbäck, 2020). Thus, lactate depletion can not only diminish cancer cell survival but also alleviate immunosuppression, potentially enhancing the effectiveness of immunotherapy. In this context, our ROS-responsive LOD@PICsomes offer a twofold therapeutic strategy: (1) prooxidation-induced cytotoxicity and (2) metabolic disruption via lactate depletion, ultimately leading to improved antitumor immunity.

Interestingly, beyond direct cytotoxicity, our findings demonstrate that the prooxidative activity of LOD@PICsomes induces immunogenic cell death (ICD), as evidenced by the upregulation of key ICD markers such as calreticulin and high-mobility group box 1 (HMGB1). The ability to trigger ICD suggests that these nanoreactors may serve as a valuable tool in combination immunotherapy, such as enhancing the response to immune checkpoint inhibitors. Given the metabolic and immunological significance of lactate within the tumor, our study highlights the potential of PICsome-based nanoreactors as a novel approach for disrupting tumor energy homeostasis while simultaneously activating immune responses against pancreatic cancer.

## 2 Materials and methods

### 2.1 Materials

ROS-responsive polycations containing thioketal (TK) linkages on the side chains—poly ([2-[[1-[(2-aminoethyl)thio]-1-methylethyl]thio]ethyl]-α,β-aspartamide) (P(Asp-ATK))—and polyanions (PEG-*b*-PAsp) were synthesized following a previously reported method (Li et al., 2020). Lactate oxidase was obtained from Toyobo Co., Ltd. Catalase, 1-ethyl-3-(3-dimethylaminopropyl) carbodiimide hydrochloride (EDC), fluorescamine, and a live/dead cell staining kit were obtained from Tokyo Chemical Industry Co., Ltd. (TCI). L-Lactate, 4-aminoantipyrine, N-ethyl-N-(2-hydroxy-3-sulfopropyl)-m-toluidine sodium salt (EHSPT), and horseradish peroxidase were sourced from Sigma-Aldrich. Alexa Fluor^®^ 647 anti-calreticulin antibody was purchased from Abcam. Cell counting kit (CCK-8) and 2′,7′-dichlorofluorescin diacetate (DCFH-DA) were purchased from Beyotime Institute of Biotechnology. Comet Assay Kit was obtained from Nanjing Jiancheng Bioengineering Institute. Proteinase K and the HMGB1 ELISA Kit were acquired from Thermo Fisher Scientific. Dulbecco’s modified Eagle medium (DMEM), fetal bovine serum (FBS), and trypsin were obtained from GIBCO. Murine pancreatic cancer cells (Panc02) were purchased from the Cell Bank of the Chinese Academy of Sciences (Shanghai, China).

### 2.2 Preparation of empty PICsomes and LOD-loaded PICsomes (LOD@PICsomes)

Stock solutions of P(Asp-ATK) (2.5 mg/mL) and PEG-*b*-PAsp (2.5 mg/mL) were prepared in 10 mM phosphate buffer (pH 7.4). For complex formation, the cationic P(Asp-ATK) solution (10 mL) was combined with the anionic PEG-b-PAsp solution (5 mL) at charge stoichiometry (equimolar amounts) and homogenized by vortex mixing (2 min). To induce crosslinking via amide bond formation, 1-ethyl-3-(3-dimethylaminopropyl)carbodiimide hydrochloride (EDC) (10 equivalents per carboxyl group) was added, and the reaction was allowed to proceed at room temperature overnight. The crosslinking density was quantified to be ∼90% by measuring the amino group content using a fluorescamine assay (Udenfriend et al., 1972).

For LOD@PICsomes preparation, varying volumes (0.3–9.6 mL; 0.3 mL, 0.6 mL, 1.2 mL, 2.4 mL, 3.6 mL, 4.8 mL, 6 mL, 7.2 mL, 8.4 mL, 9.6 mL) of LOD solution (5 mg/mL in 10 mM phosphate buffer, pH 7.4) were added to 5 mL PEG-*b*-PAsp solution, followed by addition of 10 mL P(Asp-ATK) solution. The mixtures were vortexed for complete complexation and subjected to the same crosslinking process. Unencapsulated LOD was removed through ultrafiltration using polyethersulfone membranes (MWCO of 300 kDa), with multiple wash cycles ensuring complete elimination of free LOD. The LOD@PICsomes with a LOD loading capacity of 2.6% were used in this study. The loading capacity, defined as the ratio of entrapped LOD to the total weight of LOD@PICsomes, was quantified via fluorescence spectroscopy using a standard curve method with 20 mol% Cy5-conjugated LOD as the fluorescent tracer.

### 2.3 Characterization of PICsomes and LOD@PICsomes

PICsomes and LOD@PICsomes (1 mg/mL in 10 mM phosphate buffer, pH 7.4) were prepared as previously described. To assess oxidation-triggered swelling, PICsomes (1 mg/mL, 2.3 mM thioketal groups) were treated with 25 eq. H_2_O_2_ per thioketal for 24 h. Changes in size, size distribution, and zeta potential were analyzed using dynamic light scattering (DLS) on a Malvern Zetasizer Nano ZS90, equipped with a 633 nm He-Ne laser and 173° scattering optics.

Transmission electron microscopy (TEM) imaging was performed using a JEOL-2100F electron microscope. For sample preparation, the solution was first exchanged with deionized water via ultrafiltration using a polyethersulfone membrane (MWCO of 150 kDa) to obtain clear background images. Next, 20 µL of the sample solution was applied to a copper grid, and excess liquid was gently blotted away using filter paper. The grids were then air-dried at room temperature before TEM analysis.

### 2.4 Determination of enzyme activity of the encapsulated LOD

The enzymatic activity of LOD encapsulated within PICsomes (LOD@PICsomes) was evaluated based on the cascade catalytic formation of a quinoneimine dye. Specifically, in the presence of hydrogen peroxide—generated through LOD-mediated lactate oxidation—peroxidase catalyzes the oxidative coupling of 4-aminoantipyrine and N-ethyl-N-(2-hydroxy-3-sulfopropyl)-m-toluidine sodium salt (EHSPT), resulting in the formation of the dye. In brief, a reaction mixture was prepared by combining 5 mL of 10 mM phosphate buffer (pH 7.4) containing 2.4 U/mL peroxidase, 48 mM lactate, 1.2 mM 4-aminoantipyrine, and 0.76 mM EHSPT with 0.25 mL of either LOD@PICsomes solution (0.05 mg/mL in 10 mM phosphate buffer, pH 7.4, corresponding to an LOD-equivalent concentration of 0.0013 mg/mL) or free LOD solution (0.0013 mg/mL in 10 mM phosphate buffer, pH 7.4). The mixture was incubated at 37°C for 15 min, after which 10 mL of 0.25 wt% sodium dodecyl sulfate (SDS) solution was added to terminate the reaction. The absorbance at 555 nm was then recorded using UV-vis spectroscopy. The relative enzyme activity of LOD@PICsomes was determined by comparing its absorbance to that of free LOD, with background absorbance values subtracted using blank solutions prepared under identical conditions, except that the LOD-containing solutions were replaced with 10 mM phosphate buffer (pH 7.4).

### 2.5 Evaluation of durable protective effects of vesicle structures on the encapsulated LOD

To evaluate the long-term protective effects of PICsomes on the encapsulated LOD, LOD@PICsomes and free LOD were incubated with proteinase K (1 U/mL) for 6 or 24 h in 10 mM phosphate buffer (pH 7.4) at 37°C. The enzyme activity was assessed using the above quinoneimine dye formation assay. The residual enzyme activity of LOD@PICsomes or free LOD was compared with that of the samples without proteinase K treatment to determine the stability and protective effect of PICsomes over time.

### 2.6 Cytotoxicity evaluation

Panc02 cells were plated in 96-well plates at 1 × 10^4^ cells/well in 150 μL DMEM containing 10% FBS, 100 U/mL penicillin, 100 U/mL streptomycin, and 3 mM lactate in a humidified atmosphere containing 5% CO_2_. After 24 h incubation, the medium was replaced with fresh medium, followed by the addition of LOD, LOD@PICsomes, or LOD@PICsomes with 25 equivalents of H_2_O_2_ pre-treatment for 24 h at various LOD concentrations (0.1–10 μg/mL). After 48 h incubation, the cell viability was evaluated by Cell Counting Kit 8 (CCK-8) subsequently. IC50 values were calculated by fitting the obtained dose-response curve using GraphPad Prism.

### 2.7 Evaluation of ROS generation and DNA damage and live/dead cells staining

Panc02 cells were plated in 35-mm glass-bottom culture dishes at a density of 1 × 10^5^ cells per well and incubated for 24 h. The medium contained 3 mM lactate. Subsequently, LOD@PICsomes or LOD@PICsomes combined with catalase were added at an LOD-equivalent concentration of 2.14 μg/mL, followed by a 12-hour incubation. Intracellular ROS levels were evaluated using the ROS-sensitive probe DCFH-DA, which was applied at a final concentration of 1 μM. Following a 20-minute incubation, cells were washed three times with ice-cold PBS and subsequently imaged with an Olympus IX-71 inverted fluorescence microscope.

Meanwhile, the treated cells were collected for DNA damage assessment using the Comet Assay. Briefly, Panc02 cells were pelleted and resuspended in ice-cold PBS at a concentration of 1 × 10^5^ cells/mL. A 10-μL aliquot of the cell suspension was combined with 75 μL of prewarmed (37°C) 0.7% low-melting-point agarose and layered onto a frosted slide precoated with 100 μL of 0.5% normal-melting-point agarose (prewarmed to 45°C). A coverslip was applied and maintained at 4 °C for 10 min. After coverslip removal, the slide was overlaid with an additional 75 μL of 0.7% low-melting-point agarose (prewarmed to 37 °C) and allowed to solidify for 30 min at 4°C. The slides were then immersed in prechilled lysis buffer at 4°C for 1 h, followed by PBS rinsing. Next, the slides were incubated in an alkaline electrophoresis buffer (1 mM EDTA, 300 mM NaOH) for 30 min to facilitate DNA unwinding, then subjected to electrophoresis at 25 V for 30 min. After electrophoresis, the slides were rinsed twice with 0.4 mM Tris–HCl buffer (pH 7.5). DNA was stained using 0.5 μg/mL propidium iodide and visualized under an Olympus IX-71 fluorescence microscope. DNA damage was quantified by measuring the percentage of comet tail formation.

Additionally, live and dead cells were visualized using a live/dead cell staining kit. In brief, the treated cells were incubated with Calcein-AM solution (1 µM final concentration) for 20 min to stain live cells, followed by two PBS washes. Next, PI solution was added at a final concentration of 4.5 µM and incubated for 10 min to label dead cells. After two additional PBS washes, the stained cells were observed under an Olympus IX-71 fluorescence microscope.

### 2.8 Evaluation of cytotoxicity and ROS generation ability for recycled samples

To evaluate the durable protective effect of vesicle structures on encapsulated LOD, free LOD and LOD@PICsomes at their respective IC50 concentrations (1.33 μg/mL for free LOD and 2.14 μg/mL LOD-equivalent concentration for LOD@PICsomes) were recovered and reused in cell viability and ROS generation assays according to the methods described above. The culture medium was concentrated via 50 kDa polyethersulfone ultrafiltration membranes and reapplied to fresh Panc02 cultures. This recycling protocol was repeated twice to evaluate the prolonged bioactivity of LOD@PICsomes.

### 2.9 Detection of calreticulin exposure and HMGB1 secretion

Panc02 cells were plated in 6-well plates at 1 × 10^5^ cells/well in 2 mL medium containing 3 mM lactate and incubated for 24 h. Subsequently, LOD@PICsomes or LOD@PICsomes combined with catalase were added at an LOD-equivalent concentration of 2.14 μg/mL, followed by a 12-hour incubation. The cells were detached and stained with Alexa Fluor^®^ 647 anti-calreticulin antibody (0.5 µg/10^6^ cells) for flow cytometric analysis. Meanwhile, the HMGB1 in culture supernatants were quantified by HMGB1 ELISA Kit according to the manufacturer’s protocols.

### 2.10 Statistical analysis

All data are expressed as mean ± standard deviation. Statistical analyses were conducted using GraphPad Prism 8.4. One-way analysis of variance (ANOVA) followed by Tukey’s *post hoc* test was used for comparisons. Statistical significance was defined as *P* < 0.05. Significance levels are indicated as follows: *P < 0.05, **P < 0.01, ***P < 0.001, and ****P < 0.0001.

## 3 Results

### 3.1 LOD@PICsomes nanoreactors construction

**FIGURE 2:**
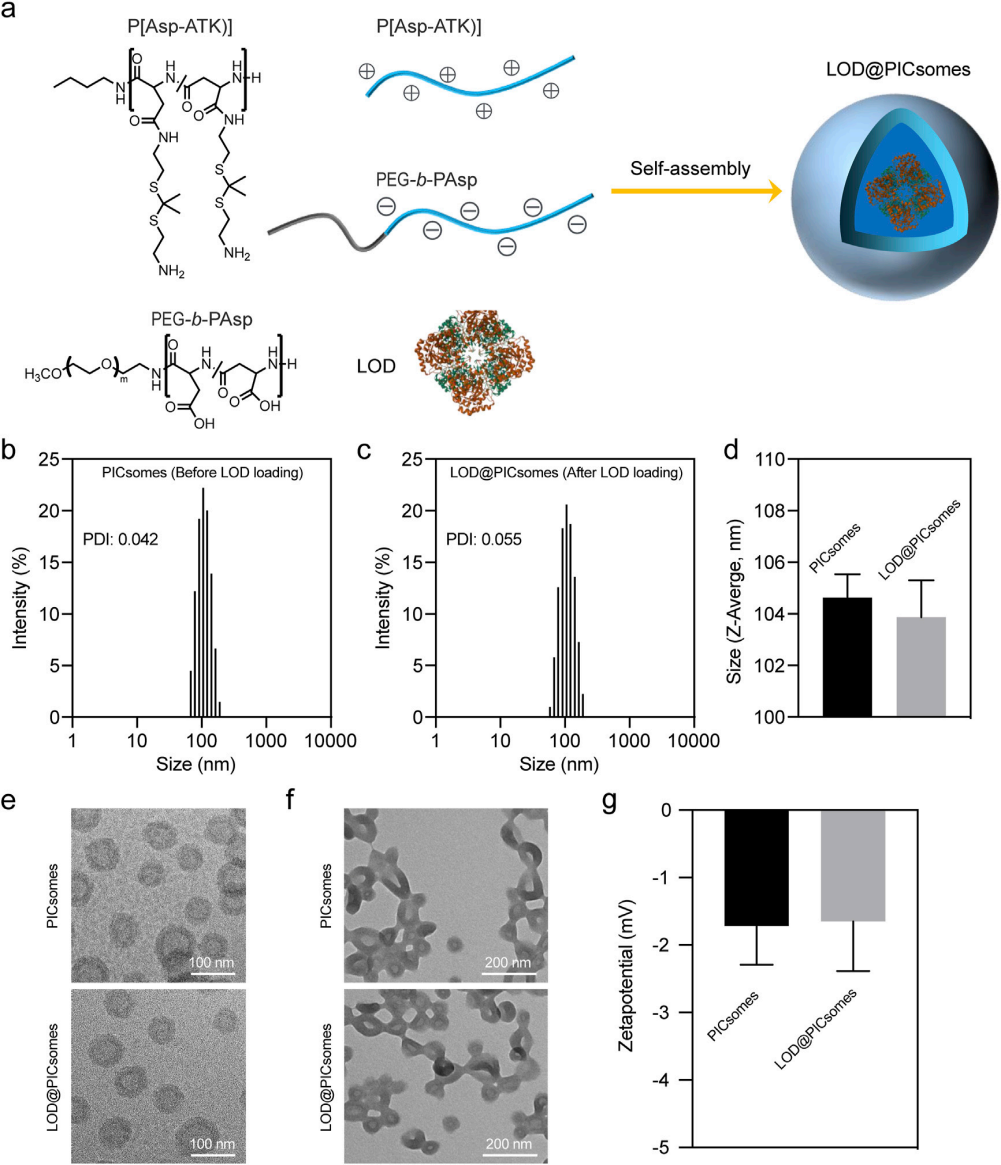
Characterization of LOD@PICsomes. **(a)** The process of electrostatic complexation of P(Asp-ATK) and PEG-*b*-PAsp in the presence of LOD forming LOD@PICsomes nanoreactors; **(b)** Intensity-weighted size distribution of empty PICsomes measured by DLS; **(c)** Intensity-weighted size distribution of LOD@PICsomes measured by DLS; **(d)** Comparison of Z-average size of empty PICsomes and LOD@PICsomes; **(e)** Cryo-TEM measurement of empty PICsomes and LOD@PICsomes. Scale bar: 100 nm; **(f)** Conventional TEM measurement of empty PICsomes and LOD@PICsomes. Scale bar: 200 nm. In conventional TEM sample preparation, the drying process disrupts the aqueous environment essential for vesicle stabilization. This leads to aggregation due to capillary forces and the loss of hydration-mediated repulsion. In contrast, cryo-TEM employs vitrification to flash-freeze samples in their native hydrated state, preserving their original morphology within a glass-like ice matrix. **(g)** Zetapotential of empty PICsomes and LOD@PICsomes measured by DLS. Data represent mean ± standard deviation (n = 3 for both **(d,g)**).

In this study, a distinct type of polymersomes, called polyion complex vesicles (PICsomes), was used for nanoreactors construction. PICsomes was constructed from electrostatic complexation of oppositely charged polyelectrolytes, amino group-bearing P(Asp-ATK) and carboxyl group-bearing PEG-*b*-PAsp (Figure 2a). Uniform PICsomes with an average diameter of ∼100 nm and a low polydispersity index (PDI) of 0.04–0.06 were successfully prepared by vortex-mixing P(Asp-ATK) and PEG-*b*-PAsp at an equimolar charge ratio for several minutes. The resulting size distribution was confirmed by dynamic light scattering (DLS) (Figure 2b). To enhance their structural integrity, the PICsomes underwent crosslinking via amidation among carboxyl and amino moieties, yielding a robust network within the vesicle membrane. The extent of crosslinking was tunable by adjusting the concentration of the coupling agent EDC, reaching a maximum of 90%. For subsequent experiments, a crosslinking density of ∼90% was chosen to maintain the long-term protection effects for sustaining the enzyme catalysis.

To construct the prooxidative and lactate-depleting nanoreactor, lactate oxidase was directly added during preparation and encapsulated within PICsomes, resulting in LOD@PICsomes. Unencapsulated enzymes were removed by ultrafiltration with multiple wash cycles using polyethersulfone membranes. DLS analysis confirmed that lactate oxidase incorporation did not alter the vesicle size or polydispersity, with LOD@PICsomes maintaining a uniform diameter of approximately 100 nm and a narrow size distribution (PDI ∼0.055) similar to empty PICsomes (Figure 2c,d). Uniform size and size distribution also indicated the efficient removal of unencapsulated enzyme. Due to the charge-matched interactions inherent in polyion complex formation, P(Asp-ATK) and PEG-*b*-PAsp preferentially combine and assemble in the presence of LOD, yielding vesicle structures without being affected by LOD (Anraku et al., 2013; Anraku et al., 2016; Li et al., 2020). Consistently, the results of TEM imaging showed a typical uniform vesicle structure before and after LOD loading (Figure 2e,f). We should mention that the aggregation of vesicles was observed for conventional TEM imaging compared to Cryo-TEM imaging. It is probably due to the drying process in conventional TEM, which disrupts the aqueous environment that stabilizes vesicle dispersion, leading to aggregation through capillary forces and the loss of hydration-mediated repulsion. In contrast, unlike conventional TEM, Cryo-TEM utilizes cryoprotection, which flash-freezes the sample in vitreous ice preserving the native state. Zetapotential measurements further demonstrated that both empty PICsomes and LOD@PICsomes had nearly neutral surface with an electrical potential around −1.7 mV (Figure 2g). These results indicated LOD loading did not influence the physicochemical properties of PICsomes including size, morphology, and surface charge.

### 3.2 Self-amplifying catalysis and long-term protection effect

**FIGURE 3:**
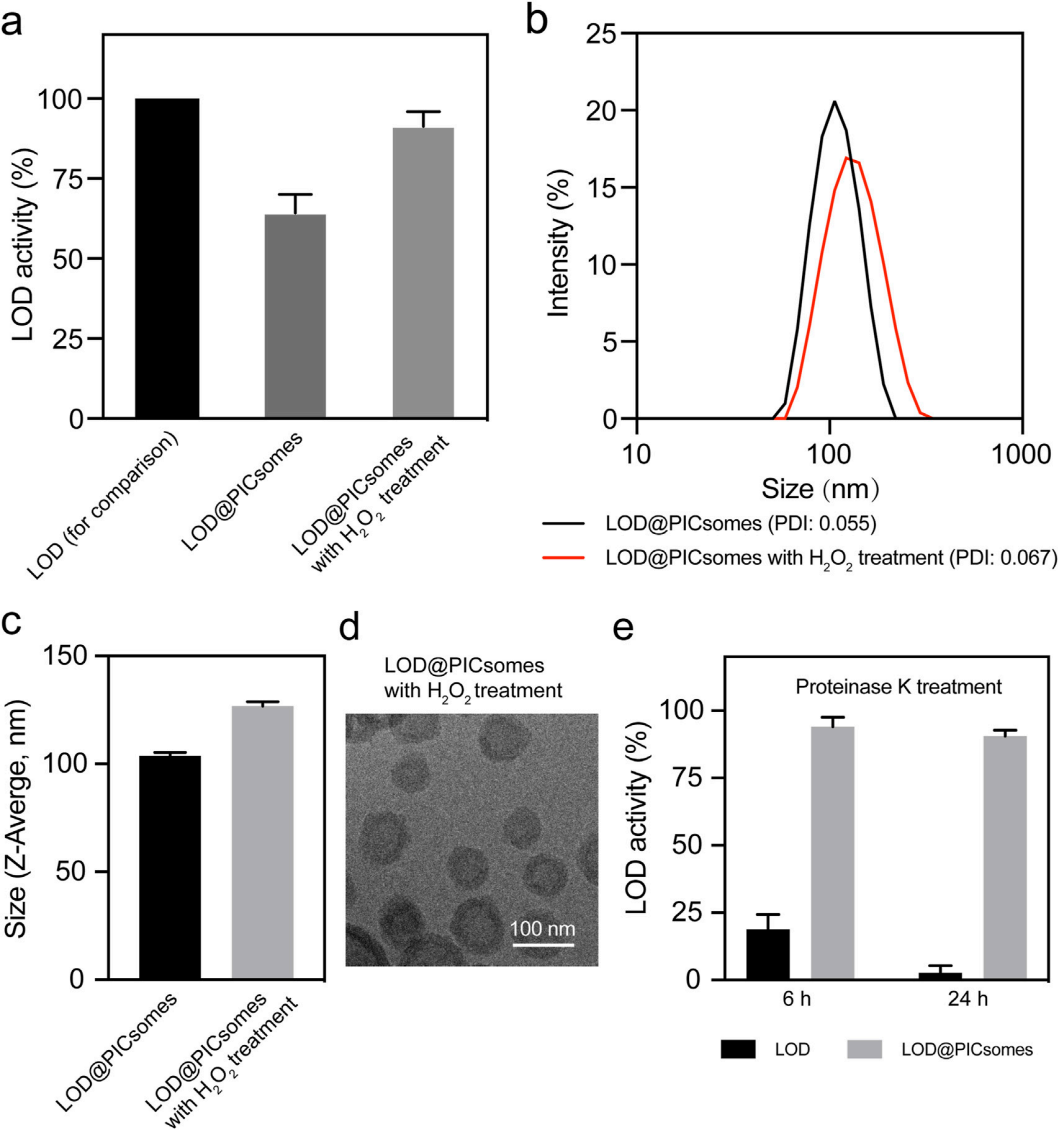
Evaluation of enzyme activity of LOD@PICsomes and swelling behavior of LOD@PICsomes. **(a)** LOD activity of LOD@PICsomes with or without H_2_O_2_ pre-treatment, with free LOD used as a control for comparison; **(b)** Intensity-weighted size distribution of LOD@PICsomes and LOD@PICsomes with H_2_O_2_ treatment measured by DLS; **(c)** Comparison of Z-average size of LOD@PICsomes and LOD@PICsomes with H_2_O_2_ treatment; **(d)** Cryo-TEM measurement of LOD@PICsomes with H_2_O_2_ treatment. Scale bar: 100 nm; **(e)** Relative enzyme activity of free LOD and LOD@PICsomes after treatment with proteinase K for 6 or 24 h. Samples (free LOD or LOD@PICsomes) without proteinase K treatment were used as controls. Data represent mean ± standard deviation (n = 3 for **(a,c,e)**).

To assess the catalytic efficiency of LOD@PICsomes, we employed the cascade reactions in the formation of quinoneimine dye as described in the Methods section. The results demonstrated that at high crosslinking densities (∼90%), LOD@PICsomes slightly decreased the enzymatic activity—∼70% to that of free LOD (Figure 3a). This suggests that the PICsome membrane with high crosslinking densities (∼90%) may limit the free exchange of substrate lactate slightly. However, after pre-treatment with LOD@PICsomes by H_2_O_2_ (25 equivalents per ROS-responsive thioketal group) for 24 h, the catalytic activity of LOD@PICsomes almost returned to the level comparable to that of free LOD. This complete activity recovery also implied that the EDC-mediated crosslinking process, which was designed to stabilize PICsomes for maintaining the long-term protection effects, did not alter enzyme structure and compromise enzyme function. It is presumably because the PICsome membrane provides a protective environment that minimizes the direct interaction of catalase carboxyl groups with EDC during the crosslinking process.

We further correlated this activity recovery with the changes in physicochemical features of PICsomes. Due to the characteristics of crosslinked membrane network, it is naturally speculated that H_2_O_2_ pre-treatment partially cleaved the crosslinks (thioketal linkages), thereby inducing swelling of PICsomes and enhancing the membrane permeability to lactate. Thioketal linkages have been extensively utilized in the design of ROS-responsive nanocarriers due to their well-established oxidative cleavage properties and excellent responsiveness to reactive oxygen species (Li et al., 2017c; Wen et al., 2025). To corroborate this, we utilized DLS to measure the size change after H_2_O_2_ pre-treatment. The results confirmed the swelling behavior, with an increase in vesicle size from 103 nm to 127 nm (Figure 3b,c). Deserved to be mentioned, even 24 h after H_2_O_2_ pre-treatment, the size distribution of LOD@PICsomes still maintained uniform as similar to the original one, indicating that H_2_O_2_ pre-treatment did not induce fragmentation of PICsomes. Cryo-TEM imaging further confirmed perfect vesicle structure after H_2_O_2_ pre-treatment (Figure 3d). The long-term capability to resist fragmentation can be ascribed to the crosslinked membrane network, in line with our previous report (Wen et al., 2025). Such resilience is crucial for maintaining the encapsulated cargo, particularly enzymes, ensuring sustained activity through membrane compartmentalization. The self-amplifying catalysis in the presence of ROS is beneficial for boosting the cytocidal effects at tumor sites with intrinsic high levels of ROS and lactate.

The combination of semipermeability, stable crosslinked membrane structures, and self-amplifying catalysis behavior facilitates the sustained lactate oxidation, ensuring long-term enzymatic performance. To further investigate the protective role of PICsomes in shielding encapsulated LOD from degradation under physiologically relevant conditions, LOD@PICsomes were exposed to proteinase K, followed by an enzymatic activity assessment. Free LOD exhibited a sharp decline in activity, losing more than 80% within the first 6 hours of incubation and retaining less than 5% activity after 24 h (Figure 3e). In contrast, LOD@PICsomes displayed remarkable stability, maintaining more than 90% enzymatic activity even after 24 h of exposure to proteinase K (Figure 3e). This robust protective effect is crucial for biomedical applications, as it not only preserves enzymatic activity but also minimizes degradation and reduces immunogenicity. These findings highlight the potential of crosslinked PICsomes as effective nanocarriers that enhance the durability and functional longevity of encapsulated enzymes, thereby enabling sustained catalytic activity in complex biological environments.

### 3.3 Cytotoxicity, cellular ROS generation, DNA damage, and retained activity of recycled LOD@PICsomes

**FIGURE 4:**
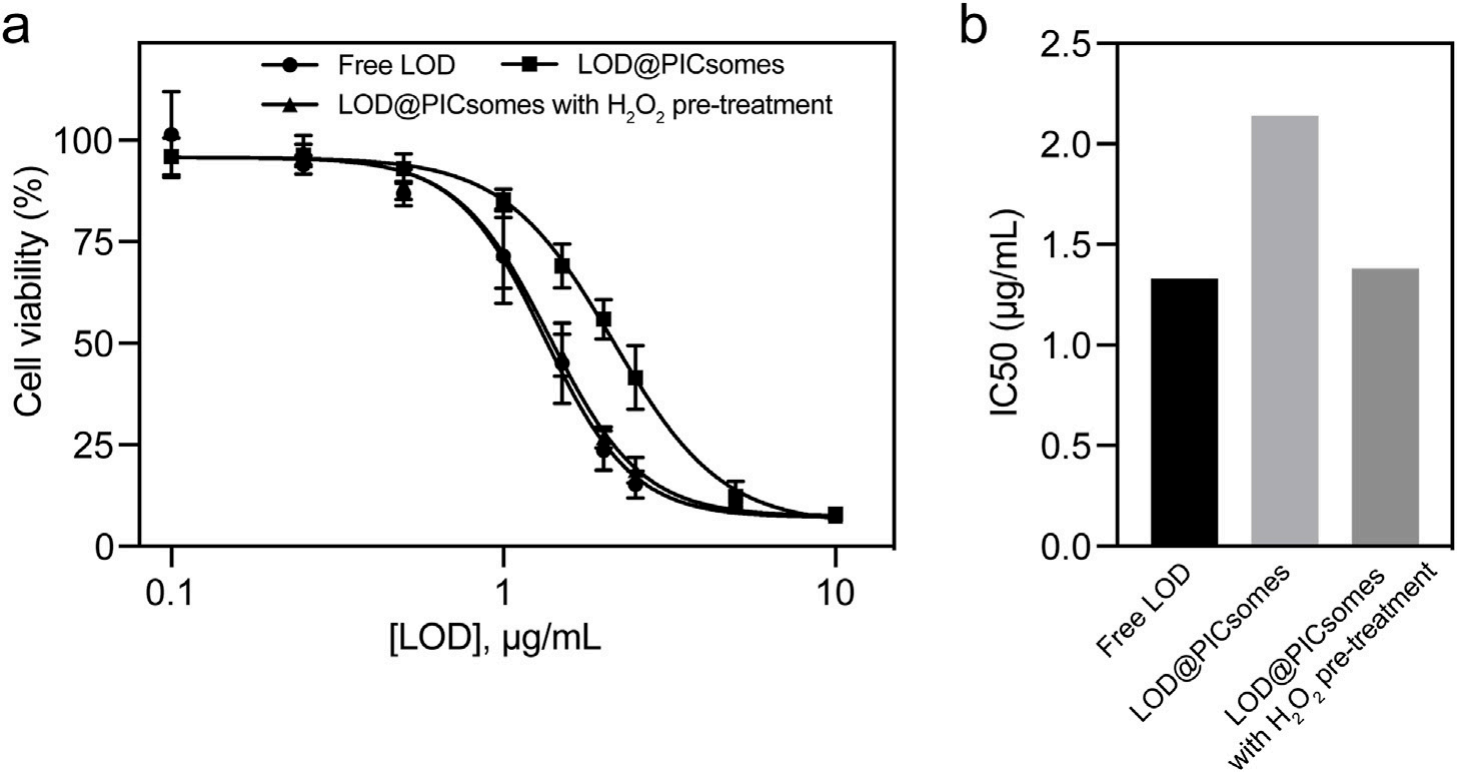
Cytotoxicity of LOD@PICsomes. **(a)** LOD concentration-dependent cytotoxicity of free LOD, LOD@PICsomes, and LOD@PICsomes with H_2_O_2_ pre-treatment against Panc02 cells; Data represent mean ± standard deviation (n = 4); **(b)** IC50 values of free LOD, LOD@PICsomes, and LOD@PICsomes with H_2_O_2_ pre-treatment, calculated using GraphPad Prism.

Next, to further evaluate the cancer-killing potential of LOD@PICsomes nanoreactors, their cytotoxicity against Pan02 cells, a well-established cell line for studying pancreatic cancer, was tested. As evidenced by dose-response curve in Figure 4a, free LOD exhibited stronger cytocidal effects than LOD@PICsomes. However, pre-treatment LOD@PICsomes with H_2_O_2_ almost made the cytocidal effects comparable to that of free LOD, which likely stems from enzyme activity recovery following ROS-induced swelling of PICsomes as demonstrated in Figure 3b. The ROS-induced swelling of the nanocarrier enhances membrane permeability, thereby facilitating improved substrate (lactate) accessibility to LOD and promoting catalytic efficiency. Quantitatively, free LOD, LOD@PICsomes, and LOD@PICsomes with H_2_O_2_ pre-treatment showed consistent IC50 values: 1.33 μg/mL, 2.14 μg/mL, and 1.38 μg/mL, respectively (Figure 4b). This enhanced cytocidal effect likely tends to occur within tumor tissues because of high levels of tumor ROS and lactate, ensuring self-amplifying catalysis for tumor specificity.

**FIGURE 5:**
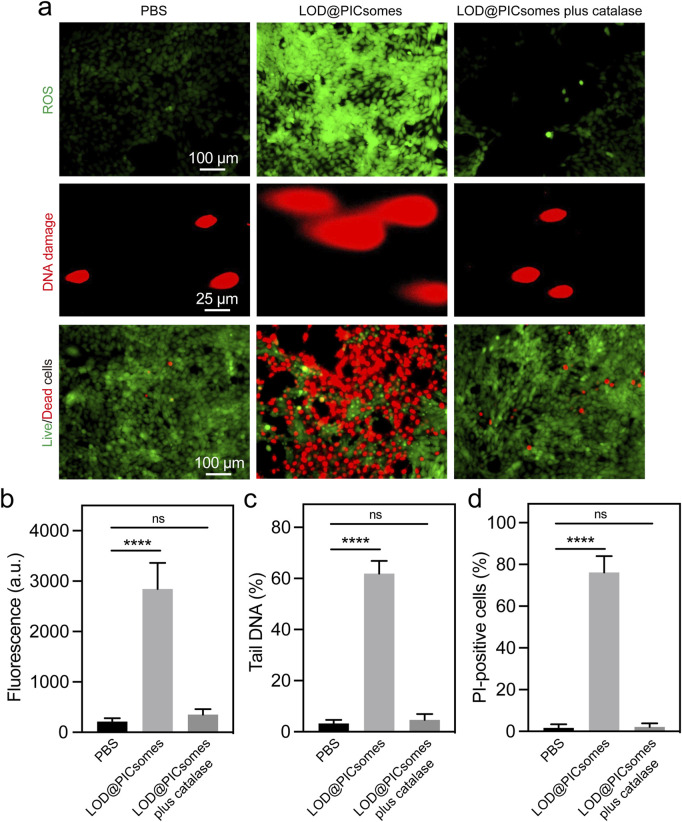
ROS generation, DNA damage, and live/dead cell assays. **(a)** ROS generation, DNA damage, and Live/dead cell staining in Panc02 cells treated with LOD@PICsomes or LOD@PICsomes plus catalase, assessed using the ROS probe DCFH-DA, comet assay, and Calcein-AM (live cell)/PI (dead cell) probes, respectively; **(b)** Quantification of ROS generation based on fluorescence intensity; **(c)** Quantification of DNA damage expressed as the percentage of tail DNA; **(d)** Quantification of the percentage of PI-positive cells (red color). The complete inactivation of LOD@PICsomes—evidenced by similar levels of intracellular ROS, DNA damage, and PI-positive cells in the ‘LOD@PICsomes plus catalase’ and PBS groups—confirms the absence of catalase toxicity. Data represent mean ± standard deviation (n = 4 randomly selected images for **(b,c,d)**. Statistical significance was assessed using one-way ANOVA followed by Tukey’s *post hoc* test.

LOD@PICsomes nanoreactors were expected to execute cytocidal effects by directly inducing oxidative damage. To investigate the underlying mechanism, we first quantified ROS generation in Pan02 cells treated with LOD@PICsomes at the IC50 concentration. As expected, LOD@PICsomes increased intracellular ROS level, as evidenced by bright fluorescence detected by DCFH-DA (Figure 5a). Quantitative fluorescence intensity showed 13-fold increase for LOD@PICsomes-treated cells compared to that of PBS group (Figure 5b). To further prove the ROS-inducing effect is directly from enzymatic generation of H_2_O_2_, the cells were treated with LOD@PICsomes plus catalase. The incorporation of catalase significantly neutralized ROS generation, demonstrating the expected source of ROS generation—H_2_O_2_, which can be efficiently decomposed by catalase (Figure 5a,b). Increased ROS can induce oxidative damage to cellular components, such as lipids, proteins, and DNA. Comet assay results revealed that 60% of LOD@PICsomes-treated cells exhibited substantial DNA damage, with a pronounced “tail” formation, compared to only 3% in the PBS-treated control group, indicating severe oxidative stress-induced DNA damage (Figure 5a,c). Similarly, the incorporation of catalase significantly rescued DNA damage, demonstrating the critical role of enzymatic generation of H_2_O_2_ in this nanoreactor (Figure 5a,c). In addition, the results of live/dead assay showed that the striking potency of LOD@PICsomes to induce cell death as evidenced by high percentage of PI-positive cells (>70%) was significantly counteracted by catalase, consistent with the experiments of ROS generation and DNA damage (Figure 5a,d). However, we would like to mention that another critical effect by this nanoreactor—lactate depletion is also expected to contribute to tumor inhibition given lactate plays a vital role in energy metabolism and immune evasion. This aspect will be clarified in future *in vivo* studies.

**FIGURE 6:**
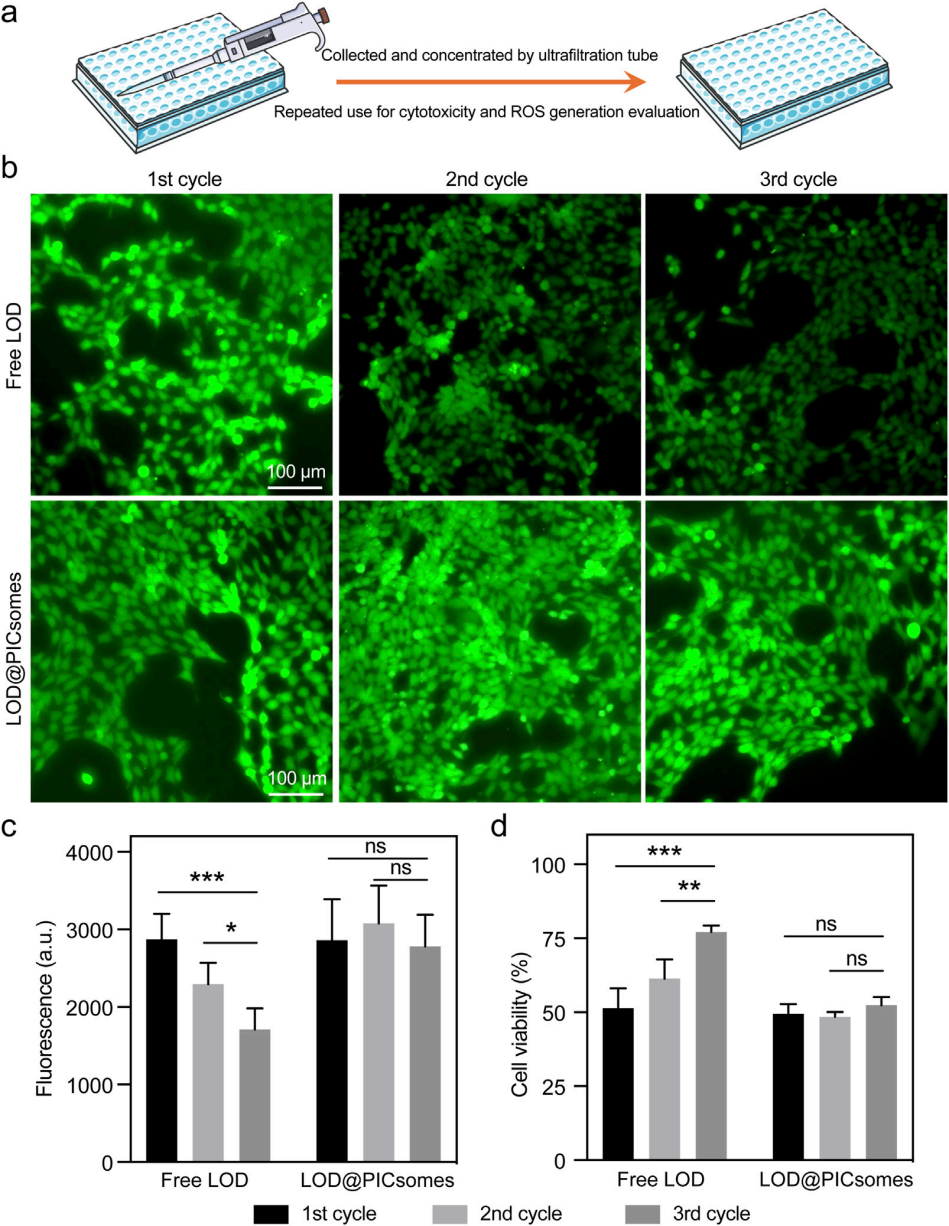
ROS generation ability and cytotoxicity and of recycled samples. **(a)** The process of recycling free LOD or LOD@PICsomes from old culture medium for repeated use in cytotoxicity and ROS generation evaluation; **(b)** ROS generation assessed using the ROS probe DCFH-DA; **(c)** Quantification of ROS generation based on fluorescence intensity in **(b)**; **(d)** Cytotoxicity of recycled samples. The concentrations used were 1.33 μg/mL for free LOD and 2.14 μg/mL LOD-equivalent for LOD@PICsomes. The samples were collected and concentrated using an ultrafiltration tube with a molecular weight cutoff (MWCO) of 50 kDa for repeated use for cytotoxicity evaluation in Panc02 cells. Data represent mean ± standard deviation (n = 4 for both **(c,d)**). Statistical significance was assessed using one-way ANOVA followed by Tukey’s *post hoc* test.

Because of the protective effect of crosslinked membrane, this nanoreactor is further anticipated to enable the sustained activity even under harsh environmental conditions. To assess this protective benefit, Pan02 cells were exposed to either free LOD or LOD@PICsomes nanoreactors at their respective IC50 concentrations. Both free LOD and LOD@PICsomes were subjected to multiple cycles of recovery and reuse in cytotoxicity evaluation. Remarkably, the recycled LOD@PICsomes nanoreactors largely retained the ROS generation and cytocidal potency even after three cycles, while free LOD progressively lost effectiveness with each cycle, as shown by decreased ROS generation ability and elevated cell viability (Figure 6). This loss is likely due to LOD degradation by proteases released from dying cells, aligning with the results from enzyme activity testing in the presence of proteinase K as showed in Figure 3e. Contrastingly, the structural stability provided by the PICsomes membrane, which shields the enzyme from degradation, enables the superior long-term performance of LOD@PICsomes.

### 3.4 Immunogenic cell death

**FIGURE 7:**
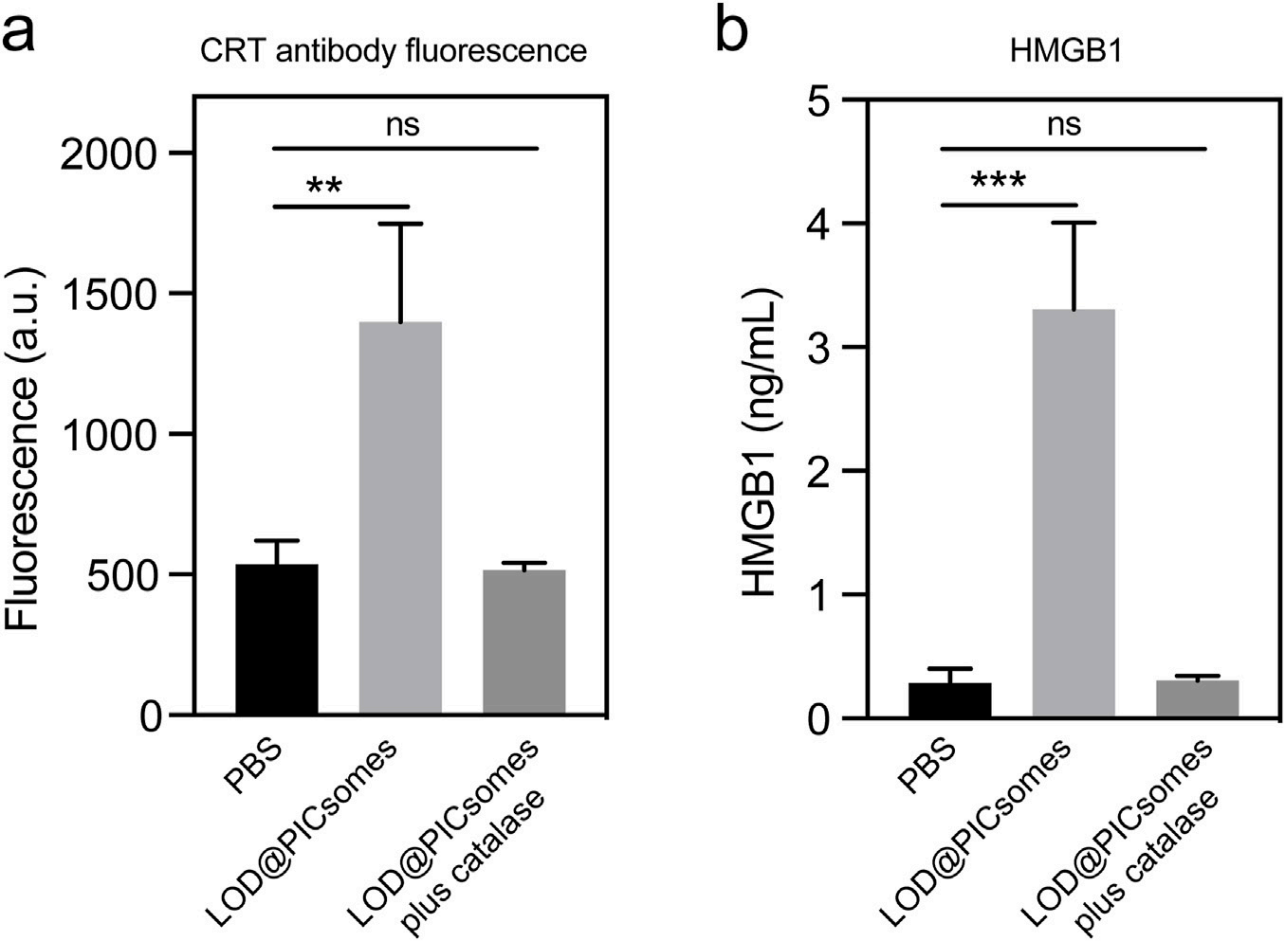
Characterization of immunogenic cell death. **(a)** Calreticulin expression in Panc02 cells treated with LOD@PICsomes or LOD@PICsomes plus catalase, analyzed using flow cytometry with an Alexa Fluor^®^ 647 anti-calreticulin antibody (mean fluorescence intensity); **(b)** HMGB1 concentration in culture supernatants of Panc02 cells treated with LOD@PICsomes or LOD@PICsomes plus catalase, measured using an HMGB1 ELISA Kit. Data represent mean ± standard deviation (n = 3 for both **(a,b)**). Statistical significance was assessed using one-way ANOVA followed by Tukey’s *post hoc* test.

ROS has the potential to induce the immunogenic cell death (ICD), releasing intracellular components as antigens and adjuvant to activate antitumor immune response. Specifically, ICD involves secretion of damage-associated molecular patterns (DAMPs), such as exposure of calreticulin (CRT) to the cell surface and release of HMGB1 to the extracellular space. We next evaluated the induction and presentation of immunogenic markers, focusing on calreticulin and HMGB1. Following treatment with the nanoreactor, flow cytometry revealed that calreticulin on the cell surface increased by 2.6 times compared to the PBS-treated control (Figure 7a). In parallel, ELISA measurements showed a substantial rise in HMGB1 levels in the culture medium, reaching 3.3 ng/mL—a marked increase compared to the minimal levels (<0.3 ng/mL) observed in the PBS-treated group (Figure 7b). Consistent with the results of ROS generation and DNA damage assays shown in Figure 5, the secretion of damage-associated molecular patterns (DAMPs) was significantly attenuated by the incorporation of catalase, as shown in Figures 7a,b. The externalization of calreticulin serves as a key “eat me” signal, actively promoting the recognition and engulfment of dying cancer cells by dendritic cells, which in turn drives their maturation. Simultaneously, the extracellular release of HMGB1 plays a crucial role in enhancing antigen processing and presentation by dendritic cells, ultimately amplifying the downstream antitumor immune response. We should also highlight that damaged DNA released into the cytosol during ICD has the potential to activate the cGAS-STING signaling pathway, thereby triggering innate immune responses. This aspect also warrants further exploration in our future investigations of this nanoreactor.

## 4 Discussion

Nanocarriers show great promise in protecting cargoes (e.g., enzymes) from degradation during transport, enhancing pharmacokinetics and biodistribution, and enabling targeted delivery (Li and Kataoka, 2021; Wen et al., 2023; Cabral et al., 2024). The development of enzyme-loaded polymer vesicles, or polymersomes, as catalytic nanoreactors represents a significant advancement in the field of nanomedicine, particularly for cancer therapy. This study introduces a class of ROS-responsive polyion complex vesicles (PICsomes) encapsulating LOD as prooxidative and lactate-depleting nanoreactors (Figure 1). These nanoreactors leverage the semipermeability and crosslinked structure of the PICsome membrane to achieve sustained enzymatic activity, while also exhibiting a self-amplifying catalytic behavior in response to elevated ROS levels. The findings presented here not only demonstrate the potential of these nanoreactors for targeted cancer therapy but also highlight their ability to induce immunogenic cell death (ICD), suggesting a promising strategy for combination immunotherapy.

One of the key challenges in the design of enzyme-loaded polymersomes is achieving a balance between maintaining structural integrity and ensuring optimal membrane permeability for sustained catalytic activity. The PICsomes developed in this study address this challenge through their crosslinked membrane structure, which provides long-term protection for the encapsulated LOD. The high crosslinking density (∼90%) ensures structural stability, while the semipermeable nature of the membrane allows for the selective exchange of small molecules, such as lactate and hydrogen peroxide (H_2_O_2_). This design enables the nanoreactors to maintain prolonged enzymatic activity, even under physiologically relevant conditions, as evidenced by the robust protection against proteinase K degradation (Figures 2,3).

The self-amplifying catalytic behavior of LOD@PICsomes is another notable feature. In response to ROS, the PICsomes undergo swelling, which enhances membrane permeability and amplifies the enzymatic catalysis of lactate, leading to increased production of H_2_O_2_. This self-amplifying mechanism is particularly advantageous in the tumor microenvironment, where elevated levels of ROS and lactate are prevalent. The resulting oxidative stress and lactate depletion contribute to the cytotoxic effects observed in pancreatic cancer cells, as demonstrated by the dose-response cytotoxicity assays (Figure 4).

The cytotoxicity of LOD@PICsomes against Panc02 pancreatic cancer cells was significantly enhanced by the self-amplifying catalysis mechanism. Pre-treatment with H_2_O_2_ restored the enzymatic activity of LOD@PICsomes to levels comparable to that of free LOD, leading to increased oxidative stress and lactate depletion within the tumor cells. This oxidative stress was further corroborated by the elevated levels of intracellular ROS, the significant DNA damage, and high percentage of PI-positive cells observed in the treated cells (Figure 5). The incorporation of catalase, which neutralizes H_2_O_2_, effectively mitigated these effects, confirming the role of enzymatic H_2_O_2_ generation in the cytotoxic mechanism. The ability of LOD@PICsomes to induce DNA damage and cell death highlights their potential as a targeted therapeutic strategy for pancreatic cancer, a malignancy known for its aggressive nature and poor prognosis. The sustained activity of the nanoreactors, even after multiple cycles of recovery and reuse, further underscores their potential for long-term therapeutic applications (Figure 6).

Beyond direct cytotoxicity, the prooxidative activity of LOD@PICsomes was shown to induce immunogenic cell death (ICD), as evidenced by the upregulation of key ICD markers such as calreticulin and HMGB1 (Figure 7). The externalization of calreticulin on the cell surface and the release of HMGB1 into the extracellular space are critical steps in the activation of antitumor immune responses. These findings suggest that LOD@PICsomes not only exert direct cytotoxic effects but also have the potential to prime the immune system for enhanced antitumor immunity (Duan et al., 2019; Wang et al., 2021; Rodrigues et al., 2022; Xie et al., 2022). The induction of ICD by LOD@PICsomes is particularly significant in the context of pancreatic cancer, which is characterized by a highly immunosuppressive tumor microenvironment (Chen et al., 2021; Xie et al., 2022). By disrupting lactate metabolism and inducing oxidative stress, these nanoreactors may help to alleviate immunosuppression and enhance the effectiveness of immunotherapy, such as immune checkpoint inhibitors. The potential activation of the cGAS-STING pathway by damaged DNA further underscores the immunogenic potential of these nanoreactors, warranting further investigation in future studies (Wang et al., 2020).

The dual therapeutic strategy of prooxidation-induced cytotoxicity and metabolic disruption via lactate depletion positions LOD@PICsomes as a promising candidate for combination immunotherapy. The ability to induce ICD and potentially activate innate immune responses suggests that these nanoreactors could be used in conjunction with other immunotherapeutic agents to enhance antitumor immunity. This approach could be particularly beneficial for pancreatic cancer, where current treatment options are limited and often ineffective.

While the results presented here are promising, further investigations are required to comprehensively evaluate the *in vivo* efficacy and safety of LOD@PICsomes. Future research should focus on evaluating the nanoreactors in animal models of pancreatic cancer, with particular attention to their biodistribution, pharmacokinetics, and potential off-target effects. Additionally, the role of lactate depletion in modulating the tumor immune microenvironment and the potential synergy with other immunotherapeutic agents should be explored.

## 5 Conclusion

In summary, this work presents a ROS-responsive enzymatic nanoreactor platform that synergistically exploits prooxidation and lactate depletion for pancreatic cancer treatment. By addressing critical challenges in enzyme-loaded polymersomes, our study contributes to the development of nanotherapeutic strategies aimed at targeting both tumor metabolism and immune evasion. These findings pave the way for future research exploring the integration of LOD@PICsomes with immunotherapies to enhance therapeutic efficacy against aggressive malignancies such as pancreatic cancer.

## Data Availability

The original contributions presented in the study are included in the article/supplementary material, further inquiries can be directed to the corresponding authors.
